# Non-Hodgkin Lymphoma of the jejunum presenting as perforation peritonitis: A case report

**DOI:** 10.1016/j.amsu.2020.04.041

**Published:** 2020-05-18

**Authors:** Warsinggih, Suluh Darmadi, Prihantono, Muhammad Faruk

**Affiliations:** a Division of Digestive, Department of Surgery, Faculty of Medicine, Hasanuddin University Makassar, Indonesia; b Division of Oncology, Department of Surgery, Faculty of Medicine, Hasanuddin University Makassar, Indonesia; c Department of Surgery, Faculty of Medicine, Hasanuddin University Makassar, Indonesia

**Keywords:** Non-Hodgkin lymphoma, Peritonitis, Intestinal perforation, Case report

## Abstract

Primary gastrointestinal lymphoma is very rare compared to gastrointestinal tract lymphoma arising from secondary to primary nodal disease. Extra nodal lymphoma can involve any part of the gastrointestinal tract, most commonly being the stomach followed by small intestine and ileocecal region. They are indistinguishable from other benign and malignant conditions and are clinically non-specific. While perforation is common among patients undergoing lymphoma treatment, presentation of primary gastrointestinal lymphoma as perforation is rare and needs proper evaluation and management. Here, we describe an interesting case in which a patient presenting with peritonitis was found to have perforation and mass of the jejunum. Resection and anastomosis were performed as intervention, with subsequent histopathological examination showing Malignant Non-Hodgkin lymphoma (NHL). Postoperative follow up was provided along with the appropriate chemotherapy regimen.

## Highlights

• Perforated hollow viscus is the perforation of any hollow viscera resulting from inflammatory, infectious, traumatic causes, and neoplasms.
• Lymphoma may cause peritonitis due to hollow viscus perforation.
• Clinicians should be aware of peritonitis due to Non-Hodgkin Lymphoma because the clinical presentation of small intestinal lymphoma is non-specific.

## 1 Introduction

Acute abdomen signify the need for prompt diagnosis and early treatment, not necessarily always surgical, pain is the main symptom and complaint [1]. The cause of acute abdomen includes perforation hollow viscus, appendicitis, volvulus and acute pancreatitis. Perforated hollow viscus is the perforation of any hollow viscera resulting from inflammatory, infectious, traumatic causes, and neoplasms [2]. Accordingly, in most cases intestinal perforation is discovered only by laparotomy and the definitive diagnosis is available only after histopathologic examination.

Lymphoma is a possible but uncommon cause of acute abdomen. The presentation of intestinal lymphoma with perforation occurs at reported frequencies of 1–25%, This perforation can be caused due to complications of chemotherapy [3].

Here, we report the uncommon case of a male suffering peritonitis with Non-Hodgkin Lymphoma located in small intestine and reported the case in accordance to the SCARE 2018 guidelines [4].

### 1.1 Case presentation

A 36-year-old male patient presented to the hospital with a 1-day history of abdominal pain on the whole abdomen. Pain was initially located at the umbilical area, but then generalized and became severe every time, and was accompanied by nausea, vomiting and fever. The patient had no history of peptic disease or trauma, but he had of significant weight loss (9 Kg over 3-months) and loos of appetite. Clinical examination revealed a heart rate of 110, respiratory rate 24 times/minute, blood pressure 140/70 mmHg, temperature 38 °C. Upon abdominal exam, we found distention of the patient's abdomen, bowel sounds were reduced on auscultation, and a tender generalized pain of the abdomen accompanied with tympanic sounds on percussion. Rectal examination was normal. Other system were normal. Complete blood count with leukocytes 14.000 cell/ml with a 75,6% neutrophil composition. Patient was negative for hepatitis B and human immunodeficiency virus (HIV). An abdominal X-ray within normal limits. We diagnosed the patient with peritonitis suspected due to perforation of the hollow viscus.

**Fig. 1:**
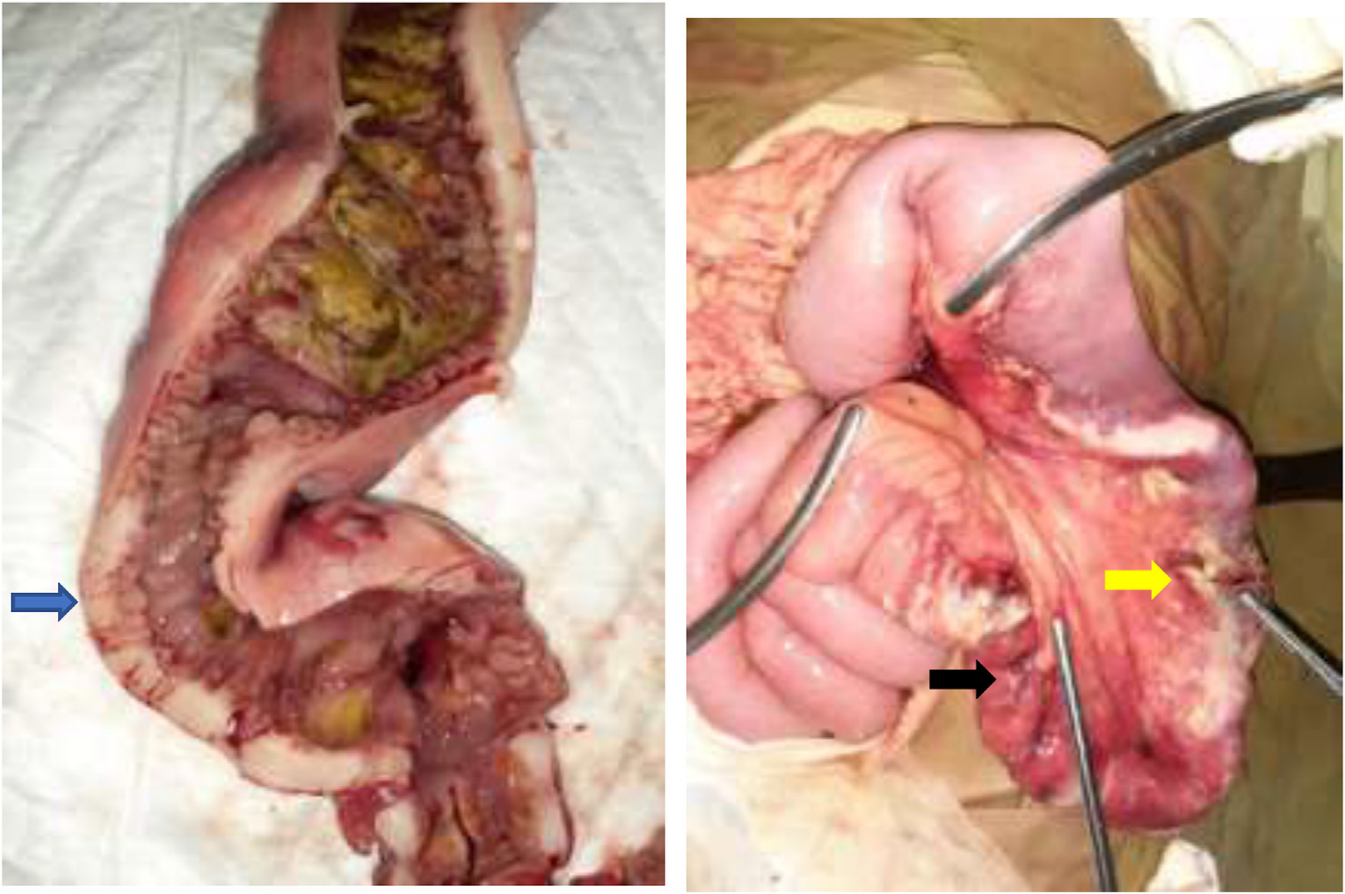
Intraoperative finding. **A**. showing the jejunal mass (blue arrow). **B**. Perforation at jejenum approximately 30 cm (yellow arrow) and 40 cm from the Treitz ligament (black arrow). (For interpretation of the references to colour in this figure legend, the reader is referred to the Web version of this article.)

An exploratory laparotomy was then performed. Immediately after the midline incision and opening of the abdomen in layers, approximately 100 ml of bowel content was revealed in the cavity. Perforation was observed on two sites within the jejunum, approximately 30 cm and 40 cm from the Treitz ligament with a size of 0,5 mm × 1 mm and 0,6 mm × 2 mm (Fig. 1). No abnormalities (tumour) were observed in the liver, spleen and peritoneum.

**Fig. 2:**
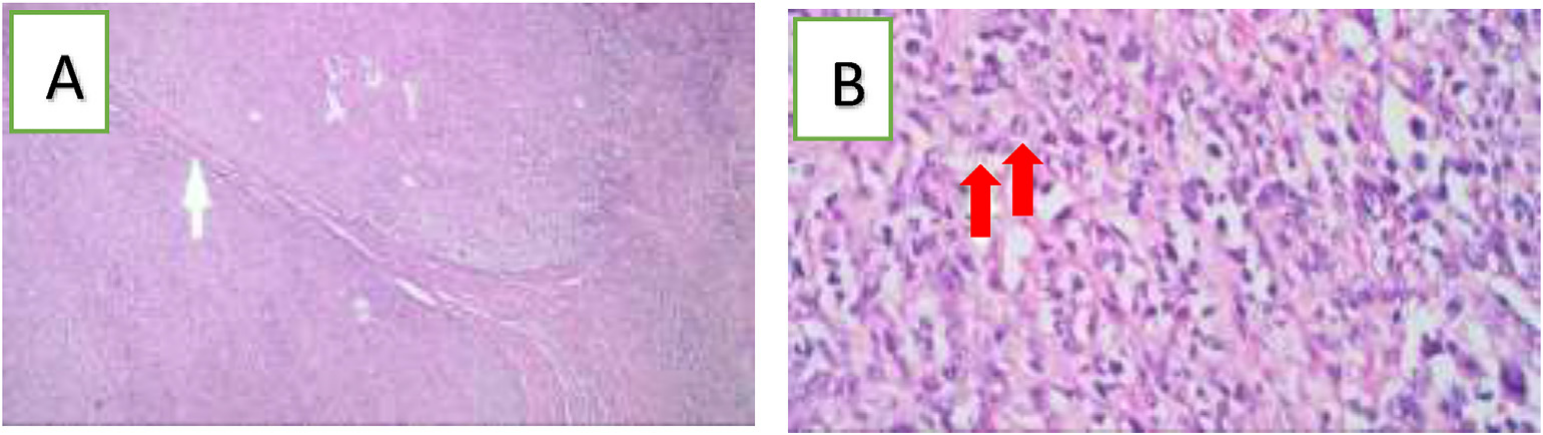
Histopathological examination showed: **A**. diffuse infiltration of the with large atypical lymphoid cells (white arrow) (hematoxylin and eosin stain 10x). **B**. Large-sized neoplastic cells with pleomorphic nuclei, variably prominent nucleoli, and scant cytoplasm. Red arrow showed mitotic cell (hematoxylin and eosin stain 100x). infiltrating all layers of the jejunal wall. (For interpretation of the references to colour in this figure legend, the reader is referred to the Web version of this article.)

**Fig. 3:**
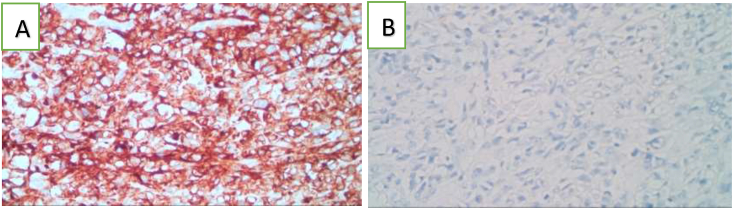
Immunohistochemical characteristics of tumor cells. **A**. CD45 immunohistochemical stain is strongly positive for neoplastic cells. **B**. CK immunohistochemical stain was negative.

We performed resection of the jejunum segment containing the mass and the mesentery, followed by end to end anastomosis. The resected jejunum specimen was sent to the anatomical pathology laboratory. Seven days after the operation, the histopathological examination of the specimens revealed features of Malignant Non-Hodgkin Lymphoma of the diffuse large B-cell type (Fig. 2) and Immunohistochemistry showed Leucocyte common antigen (CD45) positive and Cytokeratin (CK) negative on the tumour cell (Fig. 3).

The patient was discharged 8 days postoperative with a good condition, and was chemotherapy by oncology division with Cyclophosphamide-Hydroxyldaunorubicin (Doxorubicin)-VinCRIStine-Prednisone (CHOP) regimen for further management. This patients received a total of six cycles of CHOP regimen. At follow-up up to 6 months, there were no signs of recurrence on physical examination and CT scan with contrast of the chest, abdomen, and pelvis (C/A/P CT Scan) within normal limit. We doesn't performed Positron emission tomography (PET) because our institution doesn't have PET scan.

## 2 Discussion

We report the case of a 36-year-old man with primary jejunal Non-Hodgkin lymphoma (NHL), whose first clinical symptomatology were signs of peritonitis. Laparotomy performed in emergency identified a perforation in the jejunum, which required segmental resection with anastomosis.

Lymphomas of the digestive tract are uncommon, even though incidence has risen slightly in the last few decades, possibly due to improvements in immunohistochemistry which facilitates the diagnosis [5,6]. Lymphoma of gastrointestinal tract accounts for 5–10% of all case non-Hodgkin's lymphoma, with intestinal lymphoma contributing 15–20% of all gastrointestinal lymphoma [7]. Small intestinal lymphoma is predominantly in the ileum (60%–65%) followed by jejunum (20%–25%), duodenum (6%–8%) and other sites (8%–9%) [7].

The incidence, distibution of NHL subtypes, and age of presentation across geographic regions varies, it seem to be related to environmental, racial, and host [6]. Risk factors which have been found to be taking part in the pathogenesis of primary intestinal lymphoma are Coeliac disease, Helicobacter pylori infection, Campylobacter jejune, virus infection like human immunodeficiency virus (HIV), Epstein-Barr virus (EBV), Hepatitis B virus (HBV), and Human T-lymphotropic virus type 1 (HTLV-1), inflammatory bowel disease, Wegener's granulomatosis, and rheumatoid arthritis [8].

The clinical presentation of small intestinal lymphoma is non-specific and the patients have symptoms, such as non-specific abdominal pain (70–80%), weight loss (30%) [9], nausea, vomiting, loss of appetite [10] and rarely acute obstructive symptoms, intusseption, perforation or diarrhea [11]. The non-specific clinical manifestations of intestinal lymphomas make the preoperative diagnosis is difficult, so the diagnosis can be established only after surgery [10].

The definition of a primary gastrointestinal lymphoma was described by Dawson et al. (1961): [8,12].

• Absence of palpable adenopathy in clinical examination
• Absence of mediastinal lymphadenopathy in a chest x-ray
• Normal range of total and differential white cell count;
• Involvement of only regional lymph nodes, discovered on surgery;
• Liver and spleen remain without disease.

Staging of gastrointestinal lymphoma was performed according to modification the Ann Arbor staging System proposed by Musshoff [11,12] was commonly used (Table 1). Accurate staging and diagnosis of gastrointestinal lymphoma are important for the treatment [15]. However, significant improvement has been observed regarding the management of intestinal lymphoma. The World Health Organization (WHO) classifications guided by the global consensus 2008 have become widely accepted [16], and therapeutic modalities is multimodal and correlative with the histopathological type and the stage of the disease [5]. *Helicobacter pylori* eradication therapy is the first-line treatment of gastric mucosa-associated lymphoid tissue (MALT) lymphoma; involved-field radiotherapy or surgery is recommended for patients of non-gastric MALT with an early stage (I/II E); and R–CHOP regimen (rituximab, cyclophosphamide, doxorubicin, vincristine, and prednisolone) is recommended for gastric diffuse large B-cell lymphoma (DLBCL) [15,17,18].

**Table 1:** Ann Arbor lymphoma staging, modified by Musshoff [[13], [14]].

Stage of disease	Traits of stage
Stage I	Single lymphatic organ or extranodal site
Stage II	Two or more lymphatic regions on the same side of the diaphragm, or a single extranodal organ plus lymph node involvement on the same side of the diaphragm
Stage II1	Regional lymph nodes involved
Stage II2	Distant lymph nodes involved
Stage III	Lymph node involvement detected on both sides of the diaphragm
Stage IV	Disseminated disease with involvement of other extranodal sites (i.e., liver, bone marrow, abdominal wall)

The surveillance for NHL according NCCN is physical exam (looking for enlargement of node, liver and spleen), and whole body PET/CT scans with contrast of the chest, abdomen, and pelvis (C/A/P) every 3–6 months for the first 5 years, then every 12 months for every subsequent year [18]. For our patient, there were no signs of recurrence on physical examination and CT scan with contrast of the chest, abdomen, and pelvis (C/A/P CT Scan) within normal limit.

## 3 Conclusion

Morbidity and mortality increase significantly in primary gastrointestinal lymphomas presenting as perforation. However, as seen in our patient, proper understanding of the disease, timely intervention, and good clinical management will yield favourable results.

## Provenance and peer review

Not commissioned, externally peer reviewed.

## Consent

Written informed consent was obtained from the patient for publication of this case report and accompanying images.

## Author contribution

WS, SD, PRI and MF researched the literature and wrote the manuscript. WS, SD, and MF operated on the patient and had the idea for this case report. WS, and PRI checked the manuscript and made corrections. PRI and MF provided the overall guidance and support. All authors read and approved the final manuscript.

## Registration of research studies

None.

## Guarantor

Warsinggih.

## Funding

No funding or sponsorship.

## Ethical approval

The study is exempt from ethical approval in our institution.

## Declaration of competing interest

The authors declare that they have no conflict of interests.
